# Infections in the first year of living related kidney transplantation in a young transplant cohort

**DOI:** 10.1186/s12882-023-03379-9

**Published:** 2023-11-07

**Authors:** Lamis Khedr, Nahla Teama, Magdy El Sharkawy

**Affiliations:** Kidney Transplant Unit - Department of Internal Medicine - Faculty of Medicine, Ain Shams University Hospitals, Cairo, Egypt

**Keywords:** Preemptive transplant, Infection post kidney transplant, Living related kidney transplant, Tacrolimus levels

## Abstract

**Background:**

Infection after a kidney transplant is a serious cause of morbidity and mortality. Weighing the risks and benefits of immunosuppression is of paramount importance for patient wellbeing and transplant survival.

**Methods:**

This is a prospective observational study exploring the variety of bacterial, viral and fungal infections occurring within the first year of living related kidney transplantation in a young transplant cohort. Fifty-one kidney transplant recipients *(KTR)* between the age of 18 and 45 who had a kidney transplant between Jan 2020 and Jan 2022 were enrolled and followed up for one year. Primary outcome was the occurrence of infection.

**Results:**

Twenty-four patients (47%) recorded a collective 33 episodes of infection. Seven patients had repeated infections and 17 had single infections. Twenty-seven patients had an uneventful year with no infections recorded. Commonest infection was lower urinary tract infection (UTI) (27.3%) followed by SARS-COV2 and Herpes Zoster (15.2%). The commonest pathogens causing lower UTI were Escherichia coli (E coli) (21.2%) and Klebsiella (18.2%). Median Tacrolimus level was (7.8) ng/ml in *KTR* with infection and (8.95) ng/ml in *KTR* without infection, *p* = 0.21. Median Haemoglobin (IQR) was (10.2) g/dl (7.8–14) gm/dl in *KTR* with infection compared to (10.8) g/dl (7.3–15.3) in *KTR* without infection odds ratio *(OR)* = 0.78, confidence interval *(CI)* (0.5–1.1); *p* = 0.16.In *KTR* with infection 25% had donors above the age of 60 compared to 11% in *KTR* without infection ( OR 2.6,CI (0.5–12), *p* = 0.2). Post transplant diabetes *(PTDM*) occurred in (25%) in *KTR* with infection compared to those without, but that was not statistically significant p = 0. 365.In *KTR* without infection, 59.3% had a preemptive transplant compared to 20.8% in the group with infection (OR = 0.18; 95% CI: 0.052–0.631; p = 0.007). Median tacrolimus was 7.8 ng/ml in *KTR* with single infection compared to 7.7 ng/ml in *KTR* with repeated infections.

**Conclusion:**

This study shows that the commonest infection occurring in the first-year post kidney transplant was lower urinary tract infection followed by SARS-COV2 and Herpes Zoster. There was no difference in trough tacrolimus or haemoglobin levels between *KTR* who developed infection with those who did not.

**Supplementary Information:**

The online version contains supplementary material available at 10.1186/s12882-023-03379-9.

## Key learning points

What is already known about this subject?

- Kidney transplant recipients *(KTR)* are prone to infection in the first transplant year.
- Infection is a serious cause of morbidity and mortality in the transplant population.
- Lower urinary tract infections are the commonest infection early posttransplant.
- Escherichia coli is the commonest pathogen.

What does this study add?

- Infection in a young transplant cohort with living related kidney transplant seems to follow a similar pattern to older and higher immunological risk transplant patients in other studies.
- Preemptive transplant might be protective against infection.
- There was no difference in trough tacrolimus level between *KTR* who developed infection and those who did not.

What impact this may have on practice or policy?

- Preemptive transplant may offer protection against infection.

## Introduction

Infections in the first year of kidney transplantation can be quite challenging. The risk of rejection and infection need to be carefully considered on individual basis. Potential aetiologies of infection in these patients are diverse, including common community-acquired bacterial and viral diseases and uncommon opportunistic infections of clinical significance only in immunocompromised hosts [1].

The constitutional signs of infection are in many cases dampened because of the use of immunosuppression. The Recipient microbiome is changes because of surgery, immunosuppression, antibiotic prophylaxis and exposure to nosocomial and donor derived pathogens [2]. The need to diagnose and treat infection promptly is of utmost importance. Infectious exposures may promote rejection, block tolerance, or stimulate cross-reactive cellular alloimmunity [3].

In a recent study, 8.6% of kidney transplant recipients died within 5 years of transplantation and 53% of those deaths were due to infection, a rate that is twice that of the second most common cause of death [4]. Some immune monitoring strategies predict the risk of infection post transplant with some being pathogen specific and some non-specific. This depends on either quantification of peripheral blood biomarkers that reflect the status of a given arm of the immune response or the functional assessment of T cell responses [5].

In this study we explored the variety of bacterial, viral, and fungal infections occurring in the first year after kidney transplantation. This is a prospective observational study including 51 young patients transplanted from January 2020 – Jan 2022. We hypothesized that this cohort is relatively fitter than older transplant recipients which might lead to a different frequency and pattern of infections post transplant. The aim is to look at the types of infections occurring in this young transplant cohort and to assess the clinical and biochemical predictors of infection.

## Materials and methods

### Patients

The study is a prospective observational single centre study conducted in Ain Shams university Kidney Transplant Unit, Cairo between January 2020 and January 2022. Fifty one patients who received a living related kidney transplant were enrolled. We included patients aged between 18 and 45 years. Primary outcome was the occurrence of infection.

Transplantation was temporarily suspended at the height of Corona virus disease 2019 (COVID-19) pandemic. Most patients were recruited in 2021. Starting January 2021 donors and recipients received COVID vaccine.

All *KTR* in this study had full history taking and clinical examination. Immunosuppression protocol for each patient was identified according to our unit protocol. Blood tests including a full blood count, kidney function tests, urine analysis and trough tacrolimus levels were measured during clinic visits. Clinic visits were twice in the first week of transplantation weekly till the first month. Fortnightly in the second month then monthly thereafter. For *KTR* who developed an infection, additional blood test, COVID PCR,urine analysis, relevant cultures and sensitivity, imaging and trough tacrolimus level at the time of infection were obtained. If clinically indicated, additional CMV PCR was requested.

Each patient provided an informed written consent prior to enrolment. The study protocol was accepted by the Research Ethical Committee of the Faculty of Medicine-Ain Shams University. This was in accordance with the ethical guidelines of the 1975 Declaration of Helsinki.

#### Sample size

By using power analysis and Sample software (PASS 15)(Version 15.0.10)for sample size calculation, setting confidence level at 95% margin of error + -0.15. After reviewing previous study results (Saad et al. 2020) showing that (62.7%) had at least one episode of infection during follow up among patients in post renal transplant period (1 year). Based on that and after considering 10% dropout rate due to missing data, a sample size of at least 50 young kidney transplant recipients will be sufficient to achieve study objective.

### Transplant unit protocol

All patients received immunosuppression and infection prophylaxis according to the Transplant unit protocol. High immunological risk patients received 1 to 1.5 mg/kg Antithymocyte globulin *(ATG)* for 5 days. Low immunological risk patients received 20 mg Basiliximab (Day 1 and 4). All patients received 3 doses of 500 mg of Methyl Prednisolone***.*** Patients received Calcineurin Inhibitors, mostly Tacrolimus and antiproliferative agents; Mycophenolate mofetil *(MMF)* or mycophenolate sodium*(MPS) *or Azathioprine *(AZA*) for maintenance immunosuppression. Oral nystatin was given for prophylaxis against oral candida for the first month. Trimethoprim sulfamethoxazole was given for 6 months for prophylaxis against pneumocystis.

Ureteric stents were placed at the time of transplantation and removed 6 weeks later by the urologists in clinic as an outpatient procedure. Approach for ureterocystostomy was Lich Gregoir extravesical technique.

CMV D + R- and those who received ATG for induction or rejection treatment received prophylactic oral valganciclovir for 6 months. Post-surgical prophylaxis was intravenous ceftriaxone for 5 days. Starting Jan 2021 transplant donors and recipients were COVID vaccinated with 2 doses of either AstraZeneca or Sinopharm vaccine (inactivated viral vaccine).

We aimed as per protocol for tacrolimus level of 8–12 ng/ml in the first 6 months and 5–8 ng/ml for the following six months. Cyclosporin level trough (C0) zaim 200–250 ng/ml for the first 6 months and 150–200 ng/ml thereafter.

### Statistical analysis

Data were analysed using IBM SPSS software package version 20.0(Armonk, NY: IBM Corp). Qualitative data were described using number and percent. Quantitative data were described using mean ± standard deviation, median and range (minimum and maximum). Chi-square test for categorical variables, to compare between different group. Fisher’s Exact Correction for chi-square when more than 20% of the cells have expected count less than 5. Student t-test for normally distributed quantitative variables, to compare between two studied groups. Mann Whitney test for abnormally distributed quantitative variables, to compare between two studied groups. Odd ratio (OR) used to calculate the ratio of the odds and 95% Confidence Interval of an event occurring in one risk group to the odds of it occurring in the non-risk group. Significance of the obtained results was judged at the 5% level.

## Results

Fifty-one (*KTR*) were included in the study. Twenty-four patients recorded a collective 33 episodes of infection. Seven patients had repeated infections and 17 had single infections. Twenty-seven patients had an uneventful year with no infections recorded.

### Patient characteristics

This was a young transplant cohort with a mean age of 28.3 ± 10.3 yrs. Females constituted 45.1% of the cohort. The commonest cause of End Stage Renal Disease (ESRD) was unknown (33.3%), followed by hypertension (15.7%) and glomerulonephritis (13.7%).

Most patients received ATG (84.3%) for induction and the rest received Basiliximab. Most patients received tacrolimus and MMF (94.1%) for maintenance immunosuppression. Forty seven percent of *KTR* developed one or more episode of infection during the first year after transplantation.

Tacrolimus level was (7.8) ng/ml in *KTR* with infection compared to (8.95) ng/ml in *KTR* without infection (OR = 0.882;CI (0.722 – 1.077); *p* = 0.217).

There was no difference in haemoglobin level between the groups. Median Haemoglobin (IQR) was (10.2) g/dl (7.8–14) gm/dl in KTR with infection compared to (10.8) g/dl (7.3–15.3) in *KTR* without infection (OR = 0.78;CI(0.5–1.1);*p* = 0.16). PTDM occurred in (25%) in the group with infection compared to the group without, but that was not statistically significant *p* = 0.365. In the group with infection 25% of the KTR had donors above the age of 60 compared to 11% in the group without infection(OR = 2.6;CI (0.5–12); *p* = 0.2). In the group without infection, 59.3% had a preemptive transplant compared to 20.8% in the group with infection (OR = 0.18; 95% CI (0.052–0.631); *p* = 0.007) as shown in Table 1.

**Table 1:** Baseline characteristics for Kidney transplant recipients with and without infection

Baseline characteristics	Total (*n* = 51)	Kidney transplant Recipient		OR (95%C.I)	p
		**With infection (*n* = 24)**	**Without infection ( *n* = 27)**		
**Age (years)**					
Mean ± SD	28.3 ± 10.3	26 ± 9.6	30.6 ± 10.8	0.955	0.122
**Gender**					
Male	28 (54.9%)	13 (54.2%)	15 (55.6%)	0.945 (0.313 – 2.854)	0.921
Female	23 (45.1%)	11 (45.8%)	12 (44.4%)		
**Aetiology of ESKD**					
Unknown	19 (37.3%)	6 (25.0%)	13 (48.1%)	0.359 (0.109 – 1.184)	0.092
HTN	8 (15.7%)	2 (8.3%)	6 (22.2%)	0.318 (0.058 – 1.756)	0.189
GN	7 (13.7%)	3 (12.5%)	4 (14.8%)		0.760
SLE	3(5.9%)	3(12.5%)	0 (0.0%)	–	–
ADPKD	2(3.9%)	0(0%)	2 (7.4%)	–	–
VUR	2(3.9%)	2(8.3%)	0 (0.0%)	–	–
Alport	1(2%)	0(0%)	1 (3.7%)	–	–
DM type 1	1(2%)	1(4.2%)	0 (0.0%)	–	–
Preeclampsia	1(2%)	1(4.2%)	0 (0.0%)	–	–
Amyloidosis	1(4.2%)	0 (0.0%)	0 (0.0%)	–	–
CIN	2(3.9%)	2(8.3%)	0 (0.0%)	–	–
** ATG**	43(84.3%)	20(83.3%)	23 (85.2%)	0.870 (0.192 – 3.936)	0.856
**Tacrolimus Level**					
Mean ± SD	8.8 ± 3.1	8.2 ± 3.9	9.30 ± 2.0	0.882 (0.722 – 1.077)	0.217
Median (Min. – Max.)	8.8(1.4 – 19.9)	7.8(1.4 – 19.9)	8.95 (6.50 – 13.8)		
** MMF**	48(94.1%)	22(91.7%)	26(96.3%)	0.423 (0.036 – 4.985)	0.494
** Donor > 60 years**	9(17.6%)	6(25%)	3 (11.1%)	2.667 (0.586 – 12.128)	0.204
** Slow graft function**	1(2%)	0(0%)	1 (3.7%)	–	–
** PTDM**	10(19.6%)	6(25%)	4 (14.8%)	1.917 (0.469 – 7.831)	0.365
**Haemoglobin**					
Mean ± SD	10.4 ± 1.7	10.1 ± 1.7	10.7 ± 1.7	0.779 (0.550 – 1.104)	0.160
Median (Min. – Max.)	10.3(7.3 – 15.3)	10.2(7.8 – 14)	10.8(7.3 – 15.3)		
** Preemptive Tx**	21(41.2%)	5(20.8%)	16(59.3%)	0.181 (0.052 – 0.631)	0.007

*SD* Standard deviation, *C.I* Confidence interval

*OR* Odds ratio, *LL* Lower limit, *UL* Upper Limit

We compared patients with single infection to patients with repeated infection regarding tacrolimus levels. There was no difference in tacrolimus level between the two groups. Median tacrolimus was 7.8 ng/ml in the group with single infection compared to 7.7 ng/ml in patients with repeated infections as shown in Table 2.

**Table 2:** Comparison between single infections and repeated infections according to tacrolimus Level

TAC Level	Total (*n* = 33)	Single infections (*n* = 16)	Repeated infections (*n* = 17)	U	p
Median (Min. – Max.)	7.7(1.4 – 30)	7.8(1.4 – 19.9)	7.7(1.4 – 30)		

*U* Mann Whitney test

*p*: *p* value for comparing between the studied groups

Slow graft function – kidney transplants that have primary function but creatinine more than 3 mg/dl on postoperative day 3

^*^Statistically significant at *p* ≤ 0.05

### Primary site of infection

Most infections were lower urinary tract infections (27.3%) as shown in Table 3. Escherichia coli *(E coli)* (21.2%) was the commonest pathogen followed by Extended beta spectrum lactamase *(ESBL)* Klebsiella as shown in Table 4**.** Fifty percent of those patients required admission. COVID infections were the second commonest (15.2%). Starting January 2021 all transplant donors and recipients were vaccinated with 2 doses of Sinopharm (inactivated whole virus) or AstraZeneca vaccine. Four out of 5 patients with SARS-COV2 were hospitalised, one patient died, and 3 patients were admitted to hospital. None required invasive ventilation and they were discharged after an average of 14 days of hospitalization. One patient only required symptomatic treatment and home isolation.

**Table 3:** Distribution of the cases according to infections (*n* = 33)

Infections	No. (%)
**Surgical site infections**	2(6.1%)
**Urinary tract infections**	
Lower UTI	9(27.3%)
Upper UTI	3(9.1%)
**Viral Infections**	
SARS-COV2	5(15.2%)
CMV disease	2(6.1%)
Herpes zoster	5(15.2%)
** Bacterial pneumonia**	2(6.1%)
** Gastroenteritis**	3(9.1%)
** CNS Infection**	1(3%)
** Cellulitis**	1(3%)

**Table 4:** Pathogens causing Infection

Organism	Number
Unknown	1(6%)
CMV	2(6.1%)
SARS- COV 2	5(15.2%)
Ecoli	7 (21.2%)
Fungal Infection	1(3%)
Giardia	1(3%)
HSV	2(6%)
Klebsiella ESBL	4 (12.1%)
Klebsiella MDR	2 (6.1%)
Microsporum	1 (3.0%)
MRSA	1 (3.0%)
Mycoplasma	1 (3.0%)
Varicella zoster virus(VZV)	4(12.1%)

Herpes zoster comprised 15.2% of infections. Non required hospitalisation all were mild infections that recovered after a course of valaciclovir with no complications. We observed one case of CMV pneumonitis that developed during treatment of acute antibody mediated rejection and another CMV disease with fever and pancytopenia.

Three patients (5.8%) died during the study period because of sepsis due to infection with SARS-COV2 addition to CMV PCR and COVID PCR, Herpes simplex virus (HSV) encephalitis and one unknown infectious cause.

Four patients had rejection episodes during the first year. These patients all developed an infection within the first year. Infection developed around rejection time either just before rejection or during treatment of rejection.

### Timing of infection

**Fig. 1:**
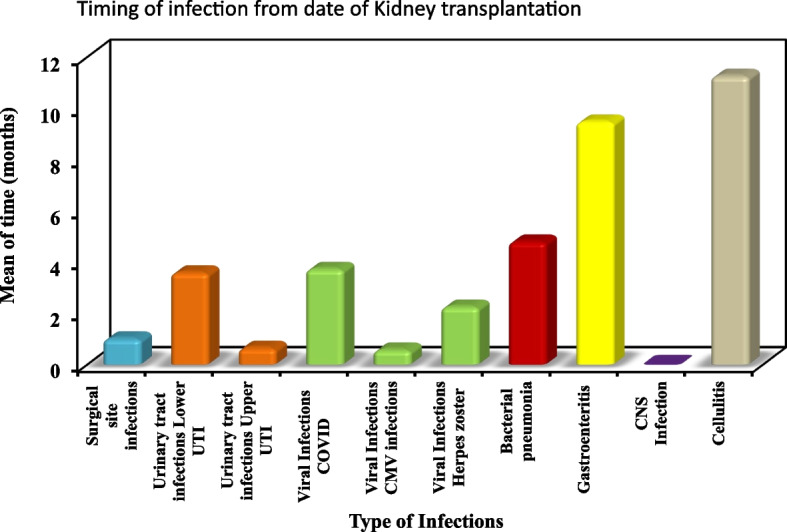
Timing of infection from date of transplantation

Most infections occurred within the first 4 months of transplant, the period of maximum immunosuppression. Surgical infections constituted 6.1% and occurred within the first month. Also, Herpes zoster appeared a median of 1.7 month after transplantation as shown in Table 5. Upper urinary tract infections median 0.47 months occurring earlier than lower Urinary tract infections median 3.3 months. Later SARS COV-2 infections appeared median 3.5 months and bacterial pneumonia median 4.8 months. Infections occurring later in the year were gastroenteritis and cellulitis as shown in Fig. 1.

**Table 5:** Timing of Infection from date of transplantation

Type of Infection	N	Min. – Max	Mean ± SD
**Surgical site infections**	**2**	0.57 – 1.43	1.00 ± 0.61
**Urinary tract infections**			
Lower UTI	**9**	0.47 – 10.97	3.59 ± 3.30
Upper UTI	**3**	0.43 – 1.03	0.64 ± 0.34
**Viral Infections**			
SARS-COV2	**5**	1.00 – 7.70	3.73 ± 2.56
CMV	**2**		0.57
Varicella zoster virus	**5**	1.30 – 4.47	2.25 ± 1.29
** Bacterial pneumonia**	2	4.03 – 5.57	4.80 ± 1.08
** Gastroenteritis**	3	0.73 – 16.93	9.48 ± 8.18
** CNS Infection**	**1**		0.0
** Soft tissue infection**	**1**		11.27

## Discussion

In this study we describe infectious complications in a young kidney transplant population during the first year after a living related kidney transplant.

This study shows that this young transplant cohort experienced similar infections to other transplant population in literature. 47% of our study population developed an infection during the first year.The commonest infections were lower urinary tract infection caused by E coli followed by Klebsiella. Infections displayed a similar pattern to those recorded in older transplant patients (40–60) and (65 +) years that was recorded in literature [6].

The second commonest infection was Varicella Zoster virus and SARS-CoV2 infections, followed by bacterial pneumonia and gastroenteritis. Varicella zoster infections were more than recorded in other studies possibly due to higher geographical prevalence and more epidemiological exposure in the population studied [7].

We found *KTR* who had a preemptive transplant had less episodes of infection than non preemptive *KTR*.The sample size was too small to draw a conclusion. This finding was mirrored in other observational trials [8]. We hypothesize that this might be related to higher haemoglobin level, less blood transfusion requirements and decreased exposure to infections when compared to the dialysis population.

There is lots of data on preferable patient and graft survival outcome in patients with preemptive kidney transplantation especially in paediatric transplant population where there is reduced risk of rejection and graft loss, but no concrete data on reduced incidence of infection. [9, 10].

Most infections occurred within the first 4 months coinciding with the time of highest immunosuppression. This state of immunosuppression was not reflected by high trough tacrolimus levels. In this study a median tacrolimus was 8.95ng/ml (6.50 – 13.8) in *KTR* with no infection and 7.8 ng/ml (1.4 – 19.9) in *KTR* with infection. In addition, there was no difference between tacrolimus levels in single and repeated infections 7.8 (1.4–19.9) for single infections and 7.7 (1.4–30) for repeated infection.

Some studies did not find high tacrolimus trough level ≥ 8 ng/mL to significantly increase the risk of infection [11, 12] this level is considered protective against acute rejection, especially during the early period after transplant. Conversely, Yun-Xia et al. found a tacrolimus level ≥ 8 ng/mL in 50% of the patients who experienced repeated infections [13].

There was no statistically significant difference in haemoglobin levels in *KTR* with no infection 10.8 g/dl (7.3 – 15.3) might suggest a protective element from infection. Whether anaemia posttransplant is considered a risk for posttransplant infections remains undetermined and requires further research and a trial with a larger cohort.

There was no difference in incidence of infection with ATG induction compared to Basiliximab. In most prospective trials, ATG was not associated with an increased risk of bacterial infection, compared with no induction therapy or other induction therapies (e.g., IL-2R antagonists and alemtuzumab) [14].

This study has certain limitations. First, it is a single centre study, which limits the generalizability of findings to other populations with different antimicrobial strategies or resources. Second, the sample size is small for accurate modelling due to the high prevalence and incidence of infections.

A trial comparing the occurrence of infection in *KTR* who had a preemptive transplant to *KTR* with non preemptive transplant is required to assess the validity of these results.

## Conclusion

This study shows that the commonest infection occurring in the first year post living related kidney transplant was lower urinary tract infection followed by SARS-COV2 and Herpes Zoster. There was no difference in trough tacrolimus, haemoglobin levels between *KTR* who developed infection and those who did not.

### Supplementary Information

**Additional file 1.**

## Data Availability

The data is available on electronic patient record database in Ain shams university kidney transplant unit and is password protected. Patients paper notes were used to retrieve some data. Data Availability The datasets are available from the corresponding author on reasonable request.
