# Supplementary material for: Infections in the first year of living related kidney transplantation in a young transplant cohort
Source: BMC Nephrol. 2023 Nov 7;24:328. doi: 10.1186/s12882-023-03379-9 (PMC10631087; doi:10.1186/s12882-023-03379-9)
Supplement: Supplementary file 1 — Additional file 1. [file 12882_2023_3379_MOESM1_ESM.pdf]

Definition of infections

Lower UTI: Clinically significant bacteriuria (> 105 CFU/ml, or > 102 CFU/ml in urine sample collected after catheter insertion), in association with symptoms of dysuria without tenderness or pain in the proximity of the transplanted kidney, with or without deterioration of graft function (1)

Upper UTI: Clinically significant bacteriuria (> 105 CFU/ml, or >102 CFU/ml in urine sample collected after catheter insertion), temperature > 38 °C and/or tenderness or pain in the proximity of the graft, and/or deterioration of renal function, blood tests showing high levels of inflammatory markers (C-reactive protein or leukocytosis) and/or renal image or biopsy compatible with pyelonephritis (1)

Pneumonia: New infiltrates on chest X-ray or computed tomography, along with three of the following: A) body temperature above 38 °C or below 36.5 °C, B) pathologic sounds to auscultation (crackles, rales, hypoventilation), C) leukocytosis or leukopenia (> 10 000 cells/ml or < 3 000 cells/ml), D) positive sputum culture or purulent sputum (> 25 leukocytes per field and an epithelial cell count <10) secondary to bacterial infection other than mycobacteria(2)

Surgical site infection: An infection that occurs in or near the surgical site within 30 days after surgery (in cases of deep surgical procedure, the period is extended past 90 days), along with purulent discharge from the site or isolation of the same microorganism in blood culture or fluid draining.(3)

Cytomegalovirus (CMV) infection: Evidence of virus replication regardless of symptoms. Although there is not a minimum preset value of viral load for considering the replication of CMV as significant, the presence of more than 5 000 copies or the increase in the number of copies by more than 1 000 in one week (4)

CMV disease: Evidence of virus replication along with symptoms such as fever, general distress, leukopenia, and thrombocytopenia, or evidence of tissue invasion (pneumonitis, hepatitis, gastrointestinal tract disease), which is defined as a positive qualitative PCR (Q-PCR) test result for tissue sample (bronchoalveolar lavage, liver biopsy, bowel biopsy, etc (4)

Cryptosporidium/ Isospora belli/ Cyclospora/ Microsporidium/ Blastocystis hominis/ Giardia-associated diarrhea: Presence of compatible clinical manifestations (usually chronic diarrhea), associated with observation of parasites or parasite eggs in stool examination (stool ova and parasites test)(5)

1. Gozdowska J, Czerwinska M, Chabros L, et al. Urinary tract infections in kidney transplant recipients hospitalized at a transplantation and nephrology ward: 1-year follow-up. Transplant Proc 2016; 48: 1580-9.

2. Qin Q, Shen KL. Community-acquired pneumonia and its complications. Indian J Pediatr 2015; 82: 745-51

3. Horan TC, Andrus M, Dudeck MA. CDC/NHSN surveillance definition of health care-associated infection and criteria for specific types of infections in the acute care setting. Am J Infect Control 2008; 36: 309-32

4. Karuthu S, Blumberg EA. Common infections in kidney trans□plant recipients. Clin J Am Soc Nephrol 2012; 7: 2058-70.

5.Kotton C, Lattes R, Practice AIDCo. Parasitic infections in solid organ transplant recipients. Am J Transplant 2009; 9: S234-S51.
